# The association between neck adiposity and long-term outcome

**DOI:** 10.1371/journal.pone.0215538

**Published:** 2019-04-23

**Authors:** Sigal Tal, Ilya Litovchik, Miriam M. Klar, Hillel S. Maresky, Noam Grysman, Itay Wiser, Itzhak Vitkon-Barkay, Gil Marcus, Oran Tzuman, David Pereg, Victoria Rum, Tomer Ziv-Baran, Shmuel Fuchs, Sa’ar Minha

**Affiliations:** 1 Department of Radiology, Assaf Harofeh Medical Center, Zerifin, Israel; 2 Sackler Faculty of Medicine, Tel-Aviv University, Tel Aviv, Israel; 3 Department of Cardiology, Assaf Harofeh Medical Center, Zerifin, Israel; 4 Department of Medical Imaging, Sunnybrook Health Sciences Center, University of Toronto, Toronto, Ontario, Canada; 5 Department of Psychiatry and Behavioral Neuroscience, Saint Louis University, St. Louis, MO, United States of America; 6 Department of Plastic and Reconstructive Surgery, Assaf Harofeh Medical Center, Zerifin, Israel; 7 Department of Cardiology, Assuta Medical Center, Ashdod, Israel; 8 Department of Cardiology, Meir Medical Center, Kfar Saba, Israel; 9 Department of Epidemiology and Preventative Medicine, School of Public Health, Sackler Faculty of Medicine, Tel Aviv University, Tel Aviv, Israel; University of Perugia, ITALY

## Abstract

Anthropometric indices of obesity (e.g. body mass index, waist circumference and neck circumference) are associated with poor long-term cardiovascular outcome. Prior studies have associated neck circumference and central body adiposity. We explored the association between neck fat volume (NFV) and long-term cardiovascular outcome. The study provides a retrospective analysis of all patients undergoing computerized tomography angiography for suspected cerebrovascular accident between January and December 2013. NFV was assessed by three dimensional reconstructions and was adjusted to height to account for differences in body sizes, thus yielding the NFV/height ratio (NHR). Univariate and multivariate analysis were utilized to explore the association between various indices including NHR and all-cause mortality. The analysis included 302 patients. The average age was 61.9±14.3 years, 60.6% of male gender. Diabetes mellitus, hypertension and cardiovascular disease were frequent in 31.5%, 69.9%, and 72.2% of patients, respectively. The median NHR was 492.53cm^2^ [IQR 393.93–607.82]. Median follow up time was 41.2 months, during which 40 patients (13.2%) died. Multivariate analysis adjusting for age, sex, and diabetes mellitus indicated an independent association between the upper quartile of NHR and all-cause mortality (hazard ratio = 2.279; 95% CI = 1.209–4.299; p = .011). NHR is a readily available anthropometric index which significantly correlated with poor long-term outcome. Following validation in larger scale studies, this index may serve a risk stratifying tool for cardiovascular disease and future outcome.

## Introduction

Obesity is global epidemic associated with increased morbidity and mortality. Defined as an abnormally high proportion of adipose tissue, obesity assessment is traditionally based on body weight, although body weight doesn’t necessarily correlate with the proportion of body fat content. Conversely, anthropometric indices describing fat distribution, such as waist-to-hip ratio, have been shown to identify patients with increased cardiovascular risk better than indices based on weight (i.e. body mass index [BMI]) [1, 2]. Furthermore, percentage of body fat is linearly correlated with mortality while this is not the case for BMI [3]. Visceral, abdominal, and liver fat, as measured by computed tomography (CT), have demonstrated associations with increased cardiovascular risk and poor outcome [4–6]. Similar to abdominal (waist) circumference, neck circumference emerged as a readily available anthropometric index, which has also demonstrated associations with cardiovascular risk factors and with poor outcome [7–11].

In a prior study, we described the methodology of calculating neck fat volume (NFV) in an unselected cohort of patients and its association with short-term outcome [12]. The current study therefore aims to analyze NFV while adjusting for height (yielding the NFV/height ratio [NHR]) and its association with long term outcome.

## Materials and methods

### Participants and data collection

The current study was a retrospective, single center analysis conducted with approval from the institutional review board at Assaf-Harofeh Medical Center, Zerifin, Israel; informed consent was waived. All consecutive patients presenting to the hospital’s emergency department between January 2013 and December 2013 who underwent computed tomography angiography (CTA) for suspected cardiovascular accident (CVA) were screened for inclusion. Excluded were patients that underwent computerized tomography (CT) for etiology other than stroke rule out (assuming these patients represent lower risk population in which all-cause mortality are less likely to be of cardiovascular etiology), those with presumed short life expectancy (i.e malignancies), and those with missing data. Demographic information including age, sex, height, weight, and concomitant medical conditions was retrospectively collected. All-cause mortality data was retrieved from the state’s Ministry of Interior database at the time of follow-up end and thus no loss of follow-up was recorded.

### Imaging protocol and analysis

CTA was performed by either Philips Brilliance iCT 256 or Philips Brilliance 64 machines (Philips, Amsterdam, the Netherlands). The scan protocol was as follows (256 MDCT protocol, and in brackets 64 MDCT): kVP 120, mAs 300 with dose modulation, slice thickness 0.9mm (1 mm), increment 0.45mm (0.5 mm), rotation time 0.5 seconds, field of view 220 mm. A bolus of 85 cc of Ultravist 370mg% (370mg iodide per 100 cc of solution, Bayer Healthcare) was injected with a rate of 5cc/sec. The technique used for NFV measurement was described and validated elsewhere.[12] Briefly, a three dimensional reconstruction of the adipose tissue of the neck (measured below the hard palate and above the angle of Louis) was performed by utilizing a window width of –150 and –30 Hounsfield units (HU), with a window center of –90 HU ideal for adipose tissue demonstration. After identification of adipose tissue, manual tracing in the different neck compartments was completed and the total volume was calculated in cubic centimeters (cm^3^). In order to adjust for variations in body sizes, we adjusted the measured NFV to the patient’s height in centimeters thus yielding the NHR (cm^3^/cm = cm^2^). Body weight or any similar anthropometric indices including weight (e.g. BMI) were excluded for adjustment to differences in NFV due to the inherent correlation between a person’s weight-reflecting total body fat and its NFV. Thus, if weight or BMI are used for adjustment- one’s weight will impact similarly on the denominator and the numerator. Height on the other hand doesn’t correlate with body weight and doesn’t change significantly during adult life. This adjustment to height was previously reported by other authors [13–16].

### Statistical analysis

Categorical variables are described as numbers and percentage. Continuous variables were evaluated for normal distribution using histogram. Normally distributed continuous variables are reported as mean and standard deviation (SD) and non-normally distributed variables are reported as median and interquartile range [IQR]. Length of follow up was evaluated using reverse censoring method.

NHR was divided into quartiles and the upper quartile (Q4) was compared to the rest of the quartiles (Q1-3). Q4 was compared to Q1-Q3 after cox regression analysis for all-cause mortality comparing each quartile to Q1 which demonstrated no statistical differences between Q1 and Q2 (p = 0.785) and between Q1 and Q3 (p = 0.986). Comparison between patients in Q4 to those in Q1-3 of NHR was performed by utilizing chi square test or Fisher’s exact test for the categorical variables while continuous variables were compared using independent samples t-test. All-cause mortality during follow up was described using Kaplan-Meier curves and comparison between patients in Q4 versus Q1-3 of NHR was performed using log-rank test. Univariate cox regression was used to evaluate the association between each variable and mortality. Variables associated with mortality at a significance level of p <0.2 alongside with sex and age were included in the multivariate analysis. The multivariate analysis included 3 blocks. The first block included NHR [Q4]. The second block included age and gender. The third block was performed using backward likelihood ratio stepwise method (p for removal > 0.1). Hazard ratios (HR) with 95% confidence intervals (CI) were recorded. All statistical tests were two-sided. P < 0.05 were considered statistically significant. SPSS was used for all statistical analysis (IBM SPSS Statistics for Windows, v.23.IBM corp.2013, Armonk, NY, USA).

## Results

**Fig 1:**
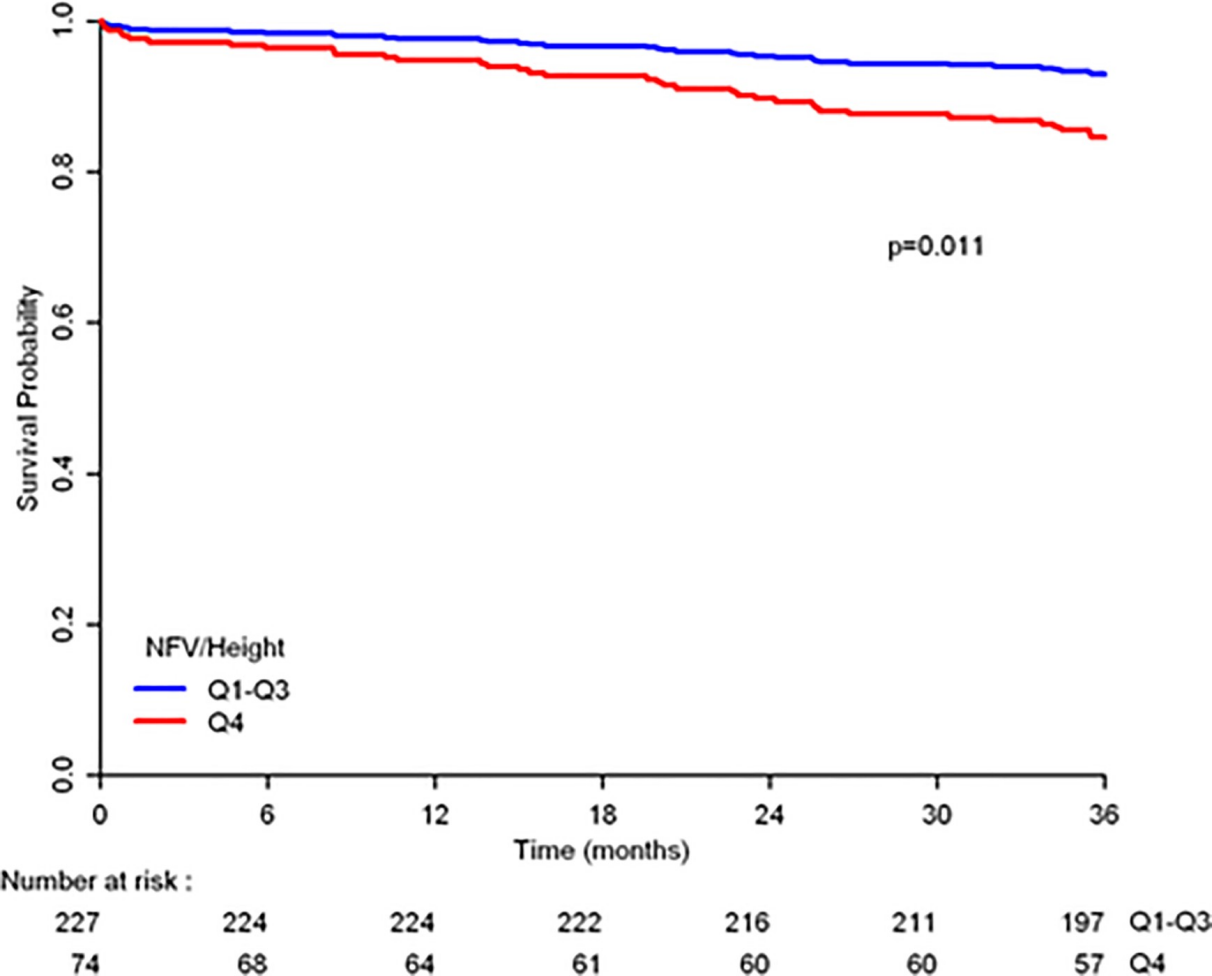
Kaplan-Meier analysis for all-cause mortality stratified by NHR quartiles.

Of the 352 patients presenting with CVA during the outlined time period, 16 were excluded due to the presence of oncological comorbidities and 34 were excluded due to missing data regarding patient height. A total of 302 patients that underwent CTA for suspected stroke were included in the final statistical analysis. Median length of follow up 41.17 months (IQR [37.6–43.97]). Mean age (±SD) of the study population was 61.9±14.3 years, 60.6% of male gender. The prevalence of diabetes mellitus, hypertension and dyslipidemia was 31.5%, 69.6% and 72.5% respectively. The mean BMI was 27.1±4.7. Patient descriptive characteristics are further described in Table 1. An NHR of 607.8 cm^2^ was found to be the value separating the lower three quartiles from the upper quartile. As detailed in Table 2, patients in the upper quartile of the NHR had higher prevalence of diabetes mellitus, hypertension, dyslipidemia, chronic kidney disease and ischemic heart disease but had a higher mean BMI (42.7% vs, 27.8%, 85.3% vs. 64.8%, 17.3% vs. 4.0%, 37.7% vs, 20.7% and 30.8±5.3 vs. 25.9±3.8 kg/m^2^ respectively; p-values<0.05 for all). During follow-up 40 patients (13.2%) expired. Kaplan-Meier survival curve (Fig 1) demonstrates poor outcome of patients in the upper quartile of NHR compared with those in the other quartiles.

**Table 1:** Study population descriptive characteristics.

Characteristic	(n = 302)
Age, years; (mean ±SD)	61.95±14.31
Male, n (%)	183 (60.6%)
Diabetes Mellitus, n (%)	95 (31.5%)
Hypertension, n (%)	211 (69.9%)
Dyslipidemia, n (%)	219 (72.5%)
Ischemic heart disease, n (%)	75 (24.8%)
Peripheral vascular disease, n (%)	18 (6.0%)
Chronic kidney disease, n (%)	22 (7.3%)
Smoking history (%)	109 (36.1%)
Body-Mass-Index; kg/m^2^ (mean ±SD)	27.1±4.7
**Neck fat and height indices**	
NFV, median (cm^3^;IQR)	823.15 (653.40–1014.88)
Height, median (cm;IQR)	168 (160–175)
NHR, median (cm^2^;IQR)	492.53 (393.93–607.82)

**Table 2:** Frequencies of co-morbidities for patients in the lower three quartiles to upper quartile of NFV/height ratio.

	NHR		
	Q1-3 (227 (75.2%))	Q4 (n = 75 (24.8%))	P value
Age; years (mean±SD)	60.73±14.53	65.70±12.97	0.009
Male, n(%)	140 (61.7%)	43 (57.3%)	0.505
Diabetes Mellitus, n(%)	63 (27.8%)	32 (42.7%)	0.016
Peripheral Vascular disease, n(%)	11 (4.8%)	7 (9.3%)	0.165
Hypertension, n(%)	147 (64.8%)	64 (85.3%)	0.001
Dyslipidemia, n(%)	158 (69.6%)	61 (81.3%)	0.049
Chronic Kidney Disease, n(%)	9 (4.0%)	13 (17.3%)	<0.001
Ischemic Heart Disease, n(%)	47 (20.7%)	28 (37.7%)	0.004
Body Mass Index; kg/m^2^ (mean±SD)	25.9±3.8	30.8±5.3	<0.001

Cox proportional hazard models exploring co-variates associated with all-cause mortality are detailed in Table 3. While univariate analysis found that age, diabetes, cardiovascular disease, hypertension and NHR were all associated with all-cause mortality, in a multivariate analysis, only age, diabetes and NHR were independently associated with this outcome. A 2.27-fold increase in the risk for mortality was recorded in patients in the upper quartile of NHR (HR = 2.279; 95% CI = 1.209–4.299; p = 0.011).

**Table 3:** Cox proportional hazard regression model with uni and multivariate analysis for co-variates associated with all-cause mortality.

	Univariate Analysis		Multivariate Analysis*	
	HR (95% CI)	p	HR (95% CI)	p
Age	1.056 (1.032–1.081)	<0.001	1.052 (1.027–1.078)	<0.001
Male	0.788 (0.423–1.470)	0.454	0.832 (0.440–1.575)	0.572
Diabetes mellitus	2.575 (1.384–4.791)	0.003	1.983 (1.051–3.743)	0.035
Peripheral vascular disease	1.861 (.662–5.229)	0.239		
Cerebrovascualr disease	2.793 (1.094–7.129)	0.032		
Hypertension	4.143(1.475–11.642)	0.007		
Dyslipidemia	1.138 (0.556–2.328)	0.723		
Chronic kidney disease	2.091 (0.819–5.337)	0.123		
Body Mass Index	1.070 (1.007–1.137)	0.029		
Ischemic Heart Disease	2.772 (1.486–5.170)	0.001		
NFV/height (Q4 vs. Q1-Q3)	2.795 (1.499–5.212)	0.001	2.279 (1.209–4.299)	0.011

*The rest of the co-variated were eliminated by the backward method.

## Discussion

The main result of the current study indicates that in an intermediate-to-high risk patient population referred to CTA for CVA exclusion, larger volume of neck adipose tissue is independently associated with long term mortality.

Obesity, one of the leading pandemics of the 21^st^ century, is known to be associated with hypertension, hyperlipidemia, diabetes mellitus and poor cardiovascular outcome [17]. Traditionally, relatively simple anthropometric indices are used to describe and correlate between adiposity and cardiovascular risk with BMI is one of the common metrics to categorized people as overweight or obese. Albeit its simple and frequent use, a better correlation with outcome was demonstrated when specific patterns of adipose tissue distribution were explored [18]. Parameters such as waist-to-hip ratio and waist circumference emerged as indices well-correlated with both cardiovascular risk and long term outcome [19–23].

Interestingly, upper body fat accumulation correlates better with these outcomes compared with fat accumulation in the lower body. This is possibly related to the fact that free fatty acid release from the upper body is higher in comparison to abdominal fat [18, 24–26]. This association between upper body fat and cardiovascular risk highlights the interest in the neck fat. The neck is a well-defined cylindrical compartment that may be assessed and measured by relatively simple means. Compared with BMI and visceral adipose tissue, neck circumference was demonstrated to correlate better with cardiovascular risk factors [8]. Furthermore, large neck circumference is associated with insulin resistance and dyslipidemia, thus clarifying the connection between this metric and cardiovascular outcome [27–29]. Increased neck circumference, however, doesn’t always indicate increase in adipose tissue volume and so actual measurement of the fat volume may be of value. This was noted in a study by Thomas et al. demonstrating in an MRI based study, that some patients have larger abdominal visceral fat than expected from their BMI [30]. They termed the acronym TOFI-” thin-on-the- outside fat-on-the-inside” to describe a phenotype for individuals at increased metabolic risk which is based on the actual visceral fat measurements and not simple anthropometric indices. The neck compartments are relatively simple to image by several modalities and was thus used for exploring the association between neck fat and cardiovascular risk. Torriani et al have demonstrated this correlation between different fat compartments in the neck and cardiovascular risk [11]. In their study, neck adipose tissue was analyzed in 303 patients that underwent positron emission tomography/CT for different etiologies. After adjusting for BMI and age, neck fat was associated with a 1.5-fold increase in the prevalence of metabolic syndrome. Neck fat was also associated with cardiovascular risk. The present study adds to the accumulating data by demonstrating an association between the upper quartile of neck-fat not only to the cardiovascular risk profile but also to poor long-term outcome.

In the initial report from our group, this association was less robust [12]. It should be noted that the aim of the previous study was to explore and detail the methodology of NFV assessment from standard CT exams. Since the present study aimed for outcome exploration, we’ve excluded patients that might have impacted both the NFV measurements (i.e. pediatric patients and those that underwent CT after trauma) and the outcome (i.e. patients with malignancies which may impact longevity). In the present study, beyond focusing in a higher-risk population, we adjusted the measured neck fat volume to height, which is believed to be a relatively constant anthropometric index and extended the follow-up period which allowed us to demonstrate the independent association with mortality.

The present study suggests that NHR may be utilized as an anthropometric metric associated with poor long-term outcome. This is in line with other anthropometric indices such as waist and neck circumference. Although the aforementioned metrics are well validated, the use of said metrics is hampered by logistical and technical issues.[31] It is thus suggested that after validation in a larger scale study, the routine assessment of NHR in patients undergoing CTA may assist in risk stratification of these patient.

The present analysis has several limitations. First, this was a relatively small cohort of subjects which limits the generalization of the conclusions. Still, the fact remains that the upper quartile of the NHR was significantly associated with poor outcome which signals for the validity of the present analysis. Second, by being a retrospective analysis including patients referred to CT in order to exclude CVA, this cohort is subjected to inherent bias. Third, the main outcome was all-cause mortality which may not necessarily indicate cardiovascular mortality.

## Conclusion

In conclusion, high NHR is independently associated with long term all-cause mortality. After validation in larger scale studies, this simple metric may be useful a screening aid to assess patients’ cardiovascular risk and long term outcome.

## Supporting information

S1 Table Dataset. (XLSX)
